# Web-Based STAR E-Learning Course Increases Empathy and Understanding in Dementia Caregivers: Results from a Randomized Controlled Trial in the Netherlands and the United Kingdom

**DOI:** 10.2196/jmir.4025

**Published:** 2015-10-30

**Authors:** Bart Hattink, Franka Meiland, Henriëtte van der Roest, Peter Kevern, Francesca Abiuso, Johan Bengtsson, Angele Giuliano, Annalise Duca, Jennifer Sanders, Fern Basnett, Chris Nugent, Paul Kingston, Rose-Marie Dröes

**Affiliations:** ^1^ VU University medical center Amsterdam Department of Psychiatry Amsterdam Netherlands; ^2^ Staffordshire University Faculty of Health Sciences Stafford United Kingdom; ^3^ Medea Firenze Italy; ^4^ Internit Lulea Sweden; ^5^ Across Limits Hamrun Malta; ^6^ University of Ulster Faculty of Computing and Engineering Newtownabbey United Kingdom; ^7^ University of Chester Department of Health & Social Care Chester United Kingdom

**Keywords:** dementia, caregivers, distance-learning, empathy

## Abstract

**Background:**

The doubling of the number of people with dementia in the coming decades coupled with the rapid decline in the working population in our graying society is expected to result in a large decrease in the number of professionals available to provide care to people with dementia. As a result, care will be supplied increasingly by untrained informal caregivers and volunteers. To promote effective care and avoid overburdening of untrained and trained caregivers, they must become properly skilled. To this end, the European Skills Training and Reskilling (STAR) project, which comprised experts from the domains of education, technology, and dementia care from 6 countries (the Netherlands, Sweden, Italy, Malta, Romania, and the United Kingdom), worked together to create and evaluate a multilingual e-learning tool. The STAR training portal provides dementia care training both for informal and formal caregivers.

**Objective:**

The objective of the current study was to evaluate the user friendliness, usefulness, and impact of STAR with informal caregivers, volunteers, and professional caregivers.

**Methods:**

For 2 to 4 months, the experimental group had access to the STAR training portal, a Web-based portal consisting of 8 modules, 2 of which had a basic level and 6 additional modules at intermediate and advanced levels. The experimental group also had access to online peer and expert communities for support and information exchange. The control group received free access to STAR after the research had ended. The STAR training portal was evaluated in a randomized controlled trial among informal caregivers and volunteers in addition to professional caregivers (N=142) in the Netherlands and the United Kingdom. Assessments were performed with self-assessed, online, standardized questionnaires at baseline and after 2 to 4 months. Primary outcome measures were user friendliness, usefulness, and impact of STAR on knowledge, attitudes, and approaches of caregivers regarding dementia. Secondary outcome measures were empathy, quality of life, burden, and caregivers’ sense of competence.

**Results:**

STAR was rated positively by all user groups on both usefulness and user friendliness. Significant effects were found on a person-centered care approach and on the total score on positive attitudes to dementia; both the experimental and the control group increased in score. Regarding empathy, significant improvements were found in the STAR training group on distress, empathic concern, and taking the perspective of the person with dementia. In the experimental group, however, there was a significant reduction in self-reported sense of competence.

**Conclusions:**

The STAR training portal is a useful and user-friendly e-learning method, which has demonstrated its ability to provide significant positive effects on caregiver attitudes and empathy.

## Introduction

The European Union (EU) is set to face major demographic challenges in the coming decades. Two main drivers for this are the (expected) doubling of the number of people with dementia and a rapid relative decline in the working population. In the Netherlands, for example, this is expected to change from a ratio of 1:42 for people with dementia to working people in 2010 to 1:16 in 2050 [1]. As a result, the task of caring for people with dementia will be provided increasingly by relatives or friends, the so-called informal caregivers, who provide this care unpaid and generally with minimal or no professional assistance. Additionally, many EU countries draft their health care policies toward an increased use of volunteers in the provision of care in addition to prolonging community-based dementia care. Therefore, to sustain and promote effective care for people with dementia, to avoid overburdening of informal and professional caregivers, and to prevent premature admission of people with dementia to long-term care settings, caregivers need to become properly skilled and feel competent in their care provision.

In an attempt to address this, e-learning interventions could prove to be a useful tool to assist informal caregivers, untrained volunteers, and professionals by offering them relevant education, training, and support [2,3] at a significantly lower cost than through face-to-face training or print distribution [4]. Interventions offered through the Internet are likely to have a lower threshold for participation given that participants can use these interventions at any time they wish, from their own homes, and with little effort. This will also help to offer access to people who would otherwise be put off by long travel times, avoid costs for visiting regular teaching sessions (eg, people living in remote areas [5]), or to people who cannot leave their home due to their caregiving role. Finally, the possibilities of the Internet allow for effective use of multimedia delivery of information (eg, graphics, animations, and interactive course material), which has been reported to enhance learning and make the material more attractive during the process of engagement [6]. Recent research has found beneficial effects from Internet-based interventions. A Cochrane review in 2005 found that “interactive health communication applications” were effective for increasing knowledge and may improve outcomes in patients and caregivers [7]. A typical means for distributing interactive health communication apps is the Internet. In another review, it was found that personalized (tailored to the individual) Internet-based interventions led to improved health in users [8].

Pilot studies offering an Internet-based program of learning for dementia caregivers found that the caregivers who evaluated it reported it as useful, educational, and convenient [9] and found positive results relating to knowledge, attitudes, self-efficacy, and empathy [10]. Among professional caregivers, e-learning was also found to be enjoyable and was reported to help acquire new skills for collaboration among professionals [11]. E-learning was also found to help staff in nursing homes to gain specific skills, such as delirium screening [12]. A review of the state-of-the-art of online course provision for providing care for people with dementia in 2011 for 4 European countries (Netherlands, the United Kingdom, Malta, and Romania) showed that 14% of the dementia courses were offered online in the Netherlands, 17% in the United Kingdom, and in both Malta and Romania there were no online courses relating to care provision for persons with dementia [13]. These findings formed the basis for the development and evaluation of a multilingual online learning platform for all types of dementia caregivers, within the EU Skills Training and Reskilling (STAR) project [14].

The European STAR project (2010-2014), funded by the European Commission in the Leonardo da Vinci Life Long Learning Programme, aimed to improve the knowledge about dementia for informal caregivers, volunteers, and professionals in dementia care by developing and evaluating an online training program in different languages and at different difficulty levels. The course content was developed from 3 theoretical perspectives: (1) the medical model of dementia, including information on types of dementia, symptoms, and diagnostics based on the *Diagnostic and Statistical Manual of Mental Disorders* (Fourth Edition, Text Revision) [15]; (2) the perspective of functional consequences in daily life based on the International Classification of Functioning, Disability and Health model from the World Health Organization [16] and how to compensate for disabilities; and (3) the perspective of dealing with the psychosocial consequences for the person with dementia and his family as described by the adaption-coping model of Dröes et al [17]. The content was composed by internationally recognized dementia experts. The platform aims to provide opportunities for collaboration, discussion, and sharing experiences between users across the EU. The main focus was to provide relevant content that was easy to find. Additionally, STAR aimed to promote accessibility to specialized knowledge by experts in the field and to offer an online community of caregivers and other stakeholders.

**Figure 1:**
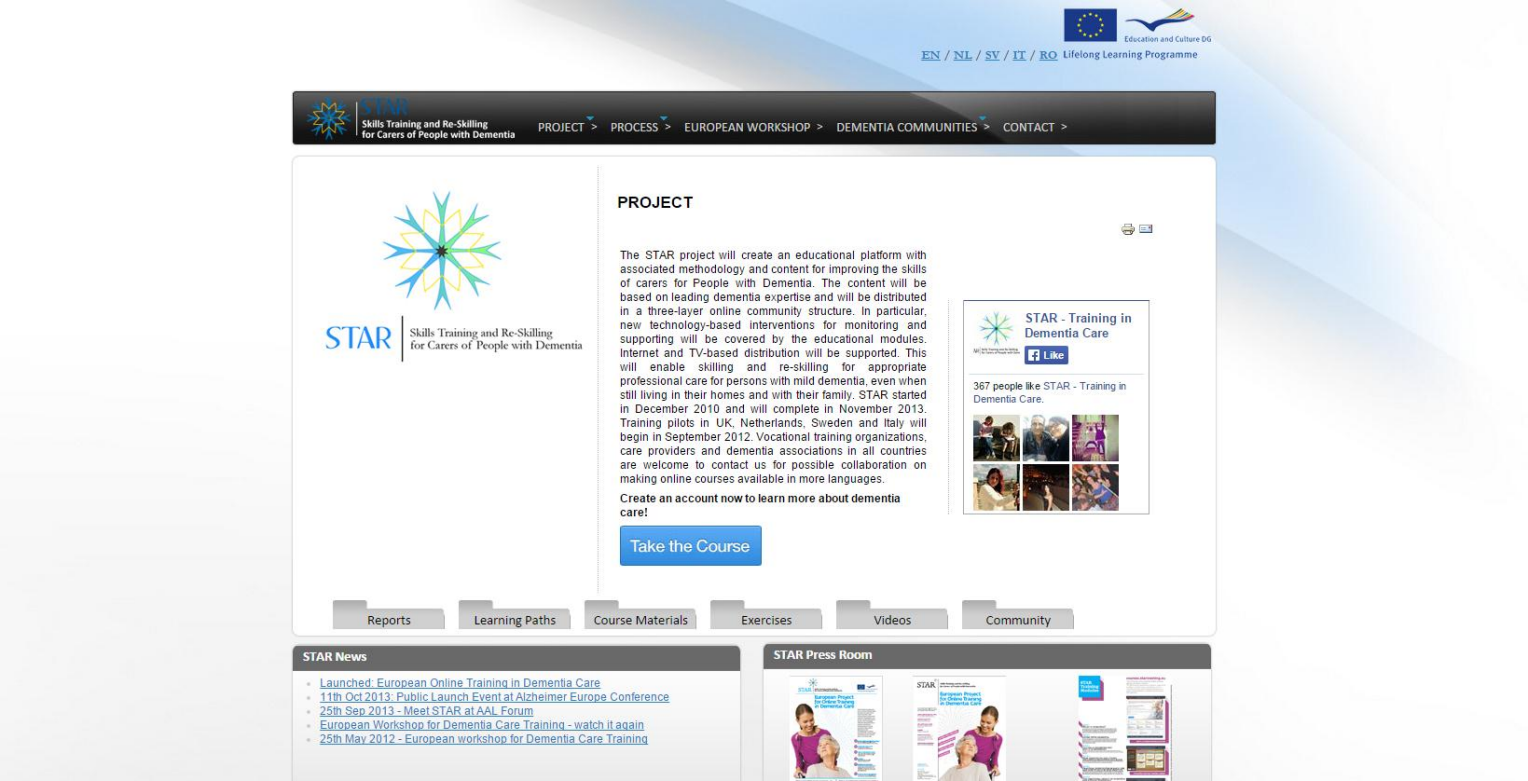
The main project page.

**Figure 2:**
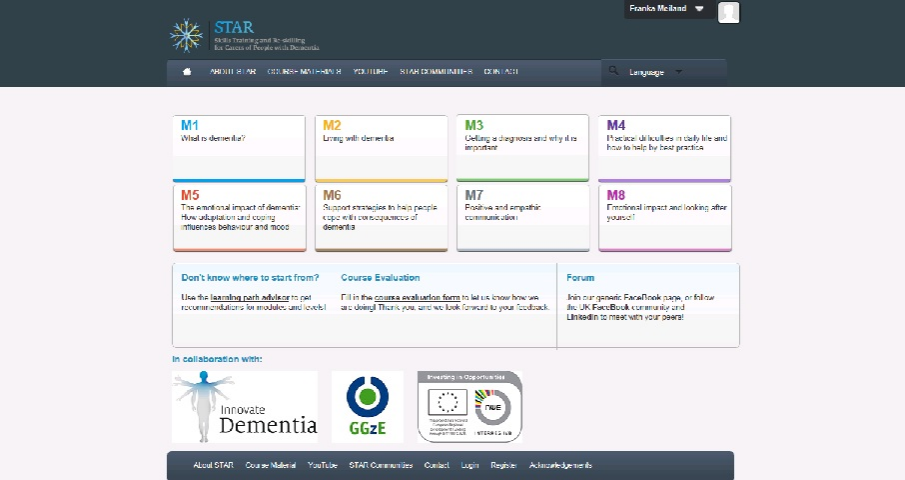
Overview of modules.

**Figure 3:**
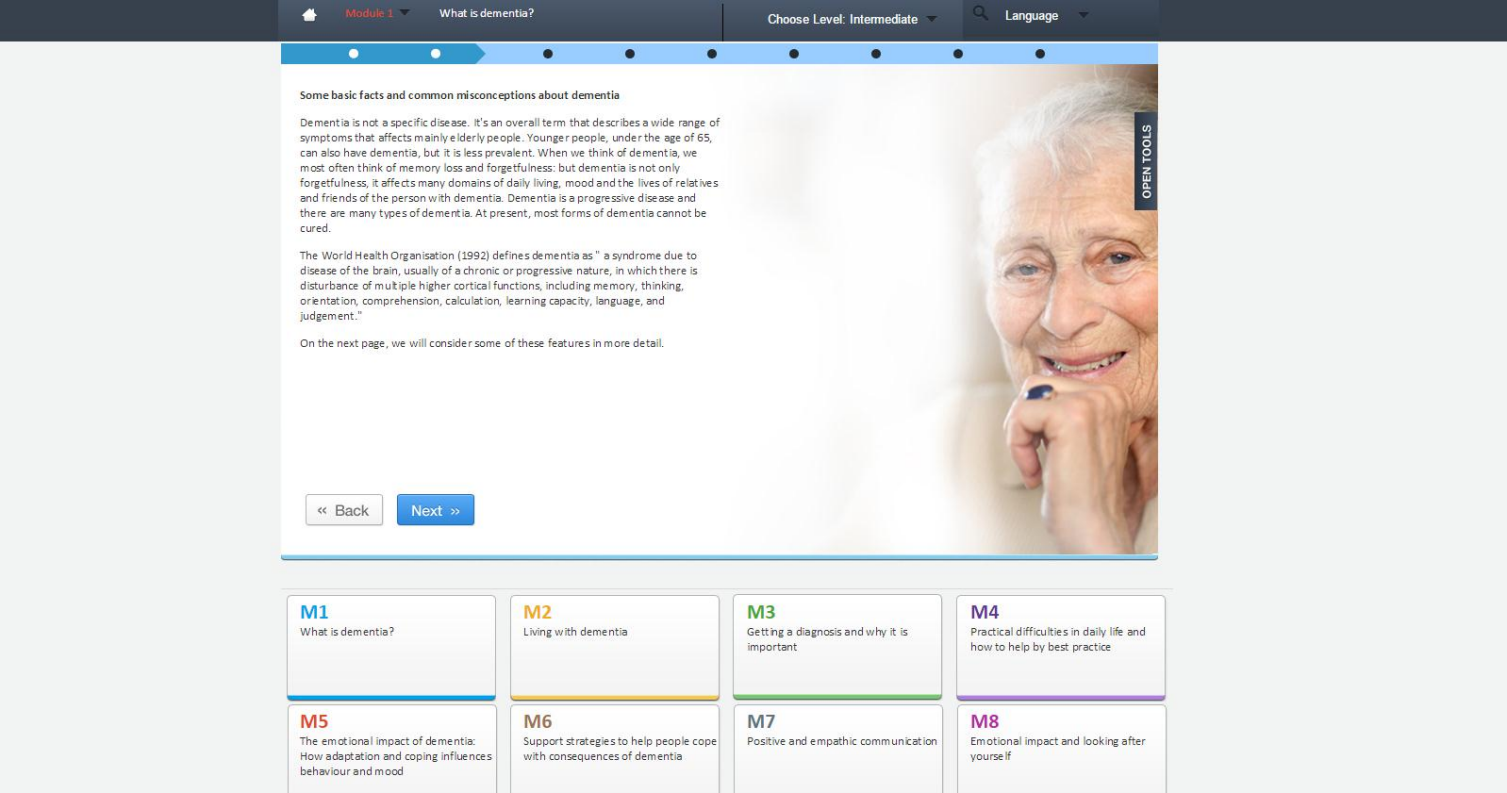
Example of a module (module 1: What is dementia?).

To this end, the STAR training portal was designed and developed (Figure 1) to offer the following functionalities:

1. A collection of 8 modules on different topics in dementia care: 2 at a basic level and 6 at an intermediate and advanced level (Figures 2 and 3);
2. A Learning Path Advisor through an online tool integrated in STAR that assesses baseline knowledge and confidence to help people decide at which point to start the course; and
3. Facebook and LinkedIn communities to promote peer support and provide opportunities to contact other dementia care professionals.

The developed course is currently available for a nominal fee. It is fully available in English and Dutch with translation into Swedish, Italian, and Romanian underway at the time of writing this paper.

After development and testing of the training portal and e-learning course material during the first phase of the STAR project, the STAR training portal was evaluated in a randomized controlled trial (RCT) in the Netherlands and the United Kingdom from May 2013 to March 2014. The primary aim of this evaluation was to assess STAR’s usefulness, user friendliness, and impact on knowledge. Because the themes of the course, in addition to factual knowledge on the dementia syndrome, focus greatly on dealing with dementia and understanding dementia (eg, themes such as “adaptation and coping,” “positive and empathic communication,” and “emotional impact and looking after yourself as a caregiver”), the impact on empathy, attitudes, and sense of competence were studied as well. The aim of this paper is to describe the results of these different types of user evaluations.

## Methods

### Design

An RCT design was used to assess the effectiveness of STAR among Dutch and English users. Participants were randomly assigned to either a group that could participate directly in the STAR training or to a group that had to wait for 4 months before they could register (free of charge) for the STAR training. Participants followed the course at their own pace; however, within a specified period of 4 months. Pretest data were gathered and follow-up data were collected after 2 to 4 months of finalizing the course in the experimental condition and after the same period in the waiting list group (control condition).

### Setting and Participants

Participants were caring for someone with dementia as an informal caregiver, a volunteer in dementia care, or a professional caregiver, and were living in either the Netherlands or in the United Kingdom. Participants in the Netherlands were recruited through meeting centers for people with dementia and their caregivers, regional branches of the national Alzheimer’s organizations, case managers, care organizations, and via announcements through several informative websites targeted at informal caregivers, volunteers, and those with an interest in dementia. In the United Kingdom, participants were recruited through caregivers’ cafes, church groups, university service users and caregiver groups, and local dementia care and welfare organizations.

Because STAR was developed both for informal caregivers (family caregivers and volunteers) as well as professional caregivers, participants who fulfilled the following criteria were recruited for the evaluation study: (1) were sufficiently computer literate to utilize the STAR website and (2) were currently an informal caregiver for someone with dementia living in the community, or a volunteer working with people with dementia with direct contact with community-dwelling people with dementia, or a professional caregiver for people with dementia with direct contact with community-dwelling people with dementia.

### The STAR Training Portal

The STAR platform was designed to be accessible through any Internet-enabled device so users could access the course at any time and place of their convenience. The STAR training portal consists of an online course with 8 modules relating to different topics. These topics were selected to cover a wide range of topics relating to dementia and dementia care. The modules consist of text, videos, interactive exercises, knowledge tests, and also include references to other websites, literature, and videos.

The themes covered in the modules are as follows:

1. What is dementia?
2. Living with dementia
3. Getting a diagnosis and why it is important
4. Practical difficulties in daily life and how to help by best practice
5. The emotional impact of dementia: how adaptation and coping influences behavior and mood
6. Support strategies to help people cope with consequences of dementia
7. Positive and empathic communication
8. Emotional impact and looking after yourself

By answering questions from an interactive “learning advisor,” participants are provided with advice relating to which module and level to start with so they may follow what may be considered a personalized learning path through the modules. For example, professionals with earlier experience in dementia care could be directed to the advanced levels of the course (including modules such as “Practical difficulties in daily life and how to help by best practice”), whereas informal caregivers who have never had to deal with dementia will be suggested to start their course with the basic modules on “What is dementia?” and “Living with dementia.” Caregivers can then follow their own learning path, either gradually working their way up to the more advanced levels or they can choose not to progress beyond the basic modules/knowledge.

To ascertain what participants learn from the modules and to make the content more appealing, interactive exercises are included in the modules. These are used after each module at the basic and intermediate levels as quizzes to test level of knowledge. If an insufficient score is achieved in the quiz, participants are encouraged to reread the material and to try the quiz again following further learning.

Participants randomly allocated to the STAR training group could follow all STAR modules and were invited to take part in their national community (communities were created for all nationalities of users) on Facebook. They were explicitly asked to follow at least 4 modules, take part in the knowledge tests offered at the end of the modules, to complete the interactive exercises during the modules, and to watch a selection of videos that were offered in the modules.

### Measurement Instruments

All questionnaires were offered online and were self-assessed in the participants’ own language. Background characteristics were inventoried for all participants. These were age, sex, relation to the person with dementia (in case of informal caregivers), time involved in care for the person with dementia, and prior experience with courses on dementia.

For assessing usefulness and user friendliness, a questionnaire was composed specifically for this study, based on the Usefulness, Satisfaction, and Ease of use (USE) questionnaire [18] and online course evaluations used by the site programmers (AcrossLimits, Malta). This questionnaire contained 29 questions with 2 open questions, 20 other questions that could be answered on a 5-point scale ranging from “strongly agree” to “strongly disagree” (eg, “I instantly knew where to click”), and 7 questions on usefulness in which participants rated the usefulness of specific parts of STAR on a 4-point scale from “very useful” to “useless.” Also, users were asked to indicate which modules they had followed and to grade each module on usefulness (1-10) to account for the fact that not all participants may have followed all modules of the course.

The primary outcome measures were knowledge on dementia and attitudes regarding dementia. Knowledge was measured with the Alzheimer’s Disease Knowledge Scale (ADKS) [19] (internal consistency α=.71, test-retest reliability=0.81). The ADKS consisted of 30 questions on different aspects of Alzheimer’s disease that could be answered with “true” or “false” (range 0-30), such as “People in their thirties can have Alzheimer’s disease.”

Attitudes toward dementia were assessed with 2 questions from the Alzheimer’s disease survey [20] and approaches to dementia with the Approaches to Dementia Questionnaire (ADQ) [21] (α=.85 for person-centered care scale; α=.76 for hope scale; α=.83 total score). The latter questionnaire was also administered among informal caregivers with one question omitted (“It is important not to become too attached to people with dementia”) because it was deemed inappropriate. The ADQ consisted of 19 questions on attitudes toward dementia and could be answered on a 5-point scale ranging from “completely agree” to “completely disagree” (range 19-95), such as “People with dementia are like children.”

The secondary outcome measures were empathy, quality of life, burden, and sense of competence. The latter 3 were only administered among informal caregivers. Empathy was assessed with the Interpersonal Reactivity Index (IRI) [22]. This questionnaire consists of 28 items that were answered on a 5-point scale ranging from “does not describe me well” to “describes me very well” and with 4 subscales: (1) perspective taking (tendency to adopt the psychological point of view of others), (2) fantasy (tendency to imagine oneself into fictitious characters in books and movies), (3) empathic concern (“other-oriented” feelings of sympathy and concern for unfortunate others), and (4) personal distress (“self-oriented” feelings of anxiety and unease in tense interpersonal settings). The range was 0 to 28 for each subscale.

Quality of life was assessed with 2 distinct questions (eg, “how would you rate your quality of life on a scale from 1 to 10?”) and burden was assessed with 1 question. Finally, for sense of competence, the Short Sense of Competence Questionnaire (SSCQ; α=.77) [23] was used. The total score for the SSCQ was calculated by dichotomizing answers to 7 questions (eg, “I feel strained in my interactions with my...”) on a 5-point scale, counting only values of 4 or 5 (range 0-7).

### Procedure

Participants, including informal caregivers (72/142, 50.7%), volunteers (24/142, 16.9%), and professional caregivers (46/142, 32.4%), in the Netherlands and United Kingdom were recruited through different partners (refer to Setting and Participants), both in person and through email. When people were interested in participating, a researcher provided them with additional written and oral information and a consent form. When a signed informed consent form was returned, the participants received a link to the online baseline questionnaire by email.

After having filled in the questionnaires, participants in the Netherlands and the United Kingdom were randomized to either the experimental or the control group. Participants were randomized based on the following variables. In each country, strata for each participant group—informal caregiver, volunteer, and professional—and within these strata (1) for informal caregivers, spouse of a person with dementia or not and knowledge regarding dementia being low (ADKS score <19), average (ADKS 20-26), or high (ADKS >27), and (2) for volunteers, shorter or longer than half a year of work experience and, for professionals, education level high or low.

Randomization software [24] was used to classify participants into either the experimental or control group. Participants in the experimental group received a link to the STAR registration webpage. People were free to choose the number of modules they followed with a baseline minimum of at least 4 to obtain a good impression of the course. People in the control group were informed that they were assigned to the group that could follow the course free of charge after post-test measurements 4 months later. At the end of the project, 2 to 4 months after the baseline measurement, all participants received a link to the questionnaires for post-test measurement. All personal data collected were anonymized. Participants were allocated a code number that was retained in a secured database under supervision of the project leaders at the evaluation sites.

### Analyses

Descriptive analyses were performed to describe the baseline characteristics of the study population. Differences between the experimental group and the control group at baseline were analyzed with relevant difference tests (chi-square and *t* tests).

The usefulness and user friendliness of the STAR training were analyzed with descriptive statistics. Impact on the outcome measures was assessed with univariate covariance analyses (ANCOVAs) on the post-test data of the participants at 4 months, whereas pretest data were included as covariates. The background variables with baseline values that differed significantly between the experimental and control group and appeared to be related to one or more of the outcome measures (ie, potential confounding variables) were also included in the analyses as covariates.

## Results

### Description of Participants

In total, 142 persons participated in the STAR evaluation study. In the Netherlands, 85 people took part in the research. Of these, 50 persons were informal caregivers, 7 were volunteers in dementia care, and 28 were professional caregivers. In the United Kingdom, 57 people participated; 22 were informal caregivers, 17 were volunteers in dementia care, and 18 were professional caregivers. We grouped the informal caregivers and volunteers together as laypeople because of the relatively small number of volunteers. The background characteristics of the participants that completed both pretest and post-test measurements are detailed in Table 1.

**Table 1:** Background characteristics of participants at baseline for laypeople (informal caregivers and volunteers) and professional caregivers that finished pretest and post-tests.

Characteristic			Experimental group	Control group	*F* (*df1,df2*)	χ^2^ (*df*)	*P*
					
**Laypeople**			n=27	n=32			
	Age (years), mean (SD)		52.93 (11.43)	54.69 (14.36)	2.02 (1,57)		.16
	**Sex, n (%)**					0.2 (58)	.65
		Male	7 (26)	10 (31)			
		Female	20 (74)	22 (69)			
	**Relationship, n (%)**					4.0 (58)	.41
		Partner	9 (33)	9 (28)			
		Child	8 (30)	5 (16)			
		Sister/brother	0 (0)	1 (3)			
		Other	4 (15)	10 (30)			
		NA	6 (22)	7 (22)			
	**Duration of care, n (%)**					7.3 (58)	.17
		<3 months	2 (7)	6 (19)			
		3-12 months	2 (7)	1 (3)			
		1-2 years	2 (7)	9 (28)			
		2-5 years	15 (58)	12 (38)			
		>5 years	6 (21)	4 (12)			
	ADKS score,^a^ mean (SD)		24.67 (3.43)	24.13 (3.32)	0.01 (1,57)		.92
**Professionals**			n=10	n=14			
	Age (years), mean (SD)		46.90 (12.12)	48.07 (9.11)	1.04 (1,22)		.32
	**Sex, n (%)**					3.1 (23)	.08
		Male	2 (20)	0 (0)			
		Female	8 (80)	14 (100)			
	**Duration of care, n (%)**					3.5 (23)	.62
		<3 months	1 (10)	2 (14)			
		3-12months	1 (10)	4 (29)			
		1-2 years	1 (10)	2 (14)			
		2-5 years	3 (30)	1 (7)			
		>5 years	4 (40)	5 (36)			
	ADKS score,^a^ mean (SD)		23.60 (3.40)	24.36 (3.52)	0.15 (1,22)		.70

^a^ADKS: Alzheimer’s Disease Knowledge Scale.

During the pilot, 59 participants dropped out. The total response at post-test was 61%. Reasons for dropouts in the Netherlands (n=29) were no time (n=4) or unknown (n=25; no response to repeated emails of researchers to remind them of filling in the questionnaires). Reasons for dropouts in the United Kingdom (n=30) were no time (n=1), no computer at home (n=1), or unknown (n=28; no response to repeated requests by researchers to fill in the questionnaires). Due to a technical issue (the rule forcing participants to fill in all usefulness and user friendliness questions before continuing did not function), one Dutch participant did not fill in the questions on usefulness and user friendliness, although he filled in all impact questions. Analyses to test differences in background characteristics between completers and dropouts indicated that for both formal and informal caregivers and volunteers there were no significant differences in age, gender, relationship, and duration of care/work between these groups. Furthermore, at baseline there were no statistically significant differences in background characteristics and primary outcome measures, such as knowledge, empathy, and approaches between the experimental and control group. For an overview of participants, refer to the flowchart in Multimedia Appendix 1. The CONSORT checklist for this study is shown in Multimedia Appendix 2.

### Results of the Evaluation of Usefulness and User Friendliness of the STAR Training

At post-test, participants from the experimental group (following the STAR training) were asked to indicate if they followed a particular module and, if so, to rate its usefulness on a scale from 0 to 10. These ratings are presented in Table 2. Participants in the Netherlands and the United Kingdom were positive overall about the usefulness of the different modules. On average across the countries, the modules that were assessed as most useful were modules 4 (practical difficulties in daily life and how to help) and 6 (support strategies to help people cope with the consequences of dementia). The modules considered least useful were modules 1 (what is dementia?) and 3 (getting a diagnosis and why it is important).

**Table 2:** Rating of usefulness of the modules by the participants at post-test (mean score on scale 1-10).^a^

Module		Laypeople, mean (SD), n		Professionals, mean (SD), n	
		Netherlands n=17	UK n=9	Netherlands n=8	UK n=2
**Baseline**					
	1. What is dementia?	8.07 (1.03), 15	8.22 (1.92), 9	8.40 (1.61), 7	6.00 (—), 1
	2 Living with dementia	8.13 (1.13), 15	9.00 (0.76), 8	8.30 (1.80), 7	—
**Intermediate**					
	3. Getting a diagnosis	7.76 (1.35), 17	8.88 (0.84), 8	7.75 (1.75), 8	—
	4. Practical difficulties	7.76 (1.30), 17	9.14 (0.69), 7	7.55 (1.75), 7	—
	5. Emotional impact of dementia	7.76 (1.56), 17	9.00 (0.82), 7	7.80 (1.67), 6	7.00 (—), 1
	6. Support strategies	7.71 (1.45), 17	8.57 (0.98), 7	8.40 (1.51), 5	9.00 (—), 1
	7. Empathic communication	7.53 (1.40), 15	9.00 (1.00), 5	8.00 (1.41), 4	10.00 (—), 1
	8. Emotional impact for caregiver	7.86 (1,41), 14	9.00 (1.00), 5	8.00 (1.41), 4	10.00 (—), 1
**Advanced**					
	3. Getting a diagnosis	7.71 (0.76), 7	6.40 (3,64), 5	6.83 (1.17), 6	—
	4. Practical difficulties	7.63 (1.19), 8	8.00 (1.00), 3	6.00 (1.00), 3	—
	5. Emotional impact of dementia	7.75 (1.04), 8	7.67 (0.58), 3	8.33 (1.53), 3	10.00 (—), 1
	6. Support strategies	7.29 (0.76), 7	8.00 (1.00), 3	7.50 (0.71), 2	—
	7. Empathic communication	7.50 (0.84), 6	7.50 (0.71), 2	7.50 (0.71), 2	—
	8. Emotional impact for caregiver	7.88 (0.84), 8	7.50 (0.71), 2	7.67, 3	—
Mean overall rating		7.74 (0.87)	8.27 (0.41)	7.16 (0.66)	8.67 (—)

^a^A higher score means participants considered it to be more useful.

The results on the opinions about usefulness of the different elements, (eg, text or videos) of the STAR training are shown in Table 3. Opinions on user friendliness are shown in Table 4.

**Table 3:** Ratings on usefulness of specific elements of the STAR training (range 1-4).^a^

Element	Laypeople, median (interquartile range)		Professionals, median (interquartile range)	
	Netherlands n=17	UK n=9	Netherlands n=8	UK n=2
The text of the modules	3.0 (0.0)	4.0 (1.0)	3.0 (1.0)	3.5 (—)
The interactive exercises	3.0 (1.0)	4.0 (1.0)	3.0 (0.0)	3.5 (—)
The knowledge questions	3.0 (1.0)	4.0 (1.0)	3.0 (1.0)	3.5 (—)
The videos	4.0 (1.0)	2.0 (2.0)	3.0 (1.0)	3.0 (—)
The online community^b^	3.0 (1.0)	3.0 (—)	4.5 (2.0)	3.0 (—)
The comments from the expert community^c^	3.0 (1.0)	3.0 (0.0)	5.0 (2.0)	3.0 (—)

^a^Scoring: 1=useless; 2=a little useful; 3=useful; 4=very useful; NA=not applicable.

^b^N/A in NL: n=8; N/A in UK: n=7.

^c^N/A in NL: n=7; N/A in UK: n=2.

**Table 4:** Opinions on user friendliness (range 1-5).^a,^ ^b^

Question	Laypeople, median (interquartile range)		Professionals, median (interquartile range)	
	Netherlands n=17	UK n=9	Netherlands n=8	UK n=2
Logging in was easy	1.0 (0.0)	1.0 (0.0)	1.0 (0.0)	2.0 (—)
I immediately noticed where I have to click	1.0 (1.0)	1.0 (0.0)	1.5 (1.0)	2.0 (—)
The overall layout is simple to follow	1.0 (1.0)	1.0 (0.0)	1.0 (1.0)	1.5 (—)
The STAR training was easy to do	1.0 (1.0)	1.0 (0.0)	1.0 (1.0)	1.5 (—)
The material has been well thought out	1.0 (1.0)	1.0 (1.0)	1.0 (0.0)	1.0 (—)
The length of the modules and exercises was just right	1.0 (1.0)	1.0 (1.0)	1.0 (1.0)	1.0 (—)
The course was nice to do	1.0 (0.0)	1.0 (1.0)	1.0 (0.0)	1.5 (—)
I knew what I had to do in the STAR training (navigating, exercises, etc)	1.0 (0.0)	1.0 (0.0)	1.0 (1.0)	1.0 (—)

^a^Scoring: 1=completely agree; 2=agree a little; 3=agree/disagree; 4=disagree a little; 5=completely disagree.

^b^In the UK cases where there's only 2 participants, the interquartile range could not be computed.

### Use of the Learning Path Advisor

Analysis of logging files of the STAR training indicated that most of the STAR participants in the experimental group used the Learning Path Advisor. In the Netherlands, 73% of informal caregivers and 91% of professional caregivers used the Learning Path Advisor to obtain a personalized suggestion where to start in the course. In the United Kingdom, 9% of informal caregivers and 17% of professionals used the Learning Path Advisor.

### Impact on Outcome Measures

In Table 5 are the results of the ANCOVA analysis to assess the impact of STAR training compared to a waiting list condition on the primary and secondary outcome measures of participants in the Netherlands and the United Kingdom.

**Table 5:** Impact of STAR training on outcome measures for laypeople and professionals (Netherlands and United Kingdom together).

Outcomes^a^				Pretest, mean (SD)		Post-test, mean (SD)		*F* (*df1*,*df2*)	*P*	η^2b^
				Experimental	Control	Experimental	Control			
**Primary outcomes**										
	**ADQ** ^c^									
		**Laypeople**		(n=27)	(n=32)	(n=27)	(n=32)			
			Total (score 18-90)	69.15 (6.74)	60.13 (10.4)	71.59 (6.48)	64.66 (4.90)	12.98 (1,57)	.001	0.19
			Hope scale (score 8-40)	20.48 (4.26)	18.25 (3.89)	22.33 (5.33)	19.13 (3.68)	3.54 (1,57)	.07	0.06
			Person scale (score 10-50)	48.67 (4.45)	41.87 (9.50)	49.26 (3.49)	45.53 (3.56)	7.48 (1,57)	.008	0.12
		**Professionals**		(n=10)	(n=14)	(n=10)	(n=14)			
			Total (score 19-95)	76.30 (5.42)	56.78 (19.6)	77.70 (5.42)	77.21 (8.12)	2.32 (1,22)	.14	0.10
			Hope scale (score 8-40)	27.50 (3.89)	21.21 (5.74)	27.80 (3.68)	27.86 (4.59)	0.31 (1,22)	.59	0.01
			Person scale (score 11-55)^d^	48.80 (2.78)	35.57 (14.4)	49.90 (3.03)	49.36 (4.41)	1.23 (1,22)	.28	0.06
	**ADKS** ^e^ **(score 1-30)**									
		**Laypeople**		(n=27)	(n=32)	(n=27)	(n=32)			
			Total	24.67 (3.43)	24.13 (3.32)	24.44 (3.11)	24.28 (3.12)	0.02 (1,57)	.90	0.00
		**Professionals**		(n=10)	(n=14)	(n=10)	(n=14)			
			Total	23.60 (3.41)	24.36 (3.52)	24.20 (2.57)	24.64 (2.40)	0.00 (1,22)	.97	0.00
	**Attitudes (score 1-7)**									
		**Laypeople**		(n=24)	(n=30)	(n=24)	(n=30)			
			Total	2.91 (1.78)	2.59 (1.82)	2.75 (1.85)	2.10 (1.67)	1.74 (1,52)	.19	0.03
		**Professionals**		(n=2)	(n=4)	(n=2)	(n=4)			
			Total	3.33 (1.32)	2.67 (1.61)	3.22 (1.39)	2.92 (1.68)	0.01 (1,4)	.93	0.00
**Secondary outcomes**										
	**Empathy (score 0-28)**									
		**Laypeople**		(n=27)	(n=32)	(n=27)	(n=32)			
			Distress	14.33 (6.20)	14.25 (5.85)	9.74 (5.33)	13.59 (5.63)	9.89 (1,22)	.003	0.15
			Empathy	12.56 (6.45)	12.81 (6.60)	20.40 (4.06)	13.03 (5.63)	47.63 (1,22)	<.001	0.46
			Fantasy	13.70 (5.19)	13.75 (4.24)	14.30 (5.24)	12.84 (4.43)	1.41 (1,22)	.24	0.03
			Perspective	13.11 (5.66)	13.06 (5.79)	18.81 (3.45)	13.75 (4.45)	25.90 (1,22)	<.001	0.32
		**Professionals**		(n=10)	(n=14)	(n=10)	(n=14)			
			Distress	13.30 (7.69)	13.86 (7.68)	7.50 (2.80)	14.57 (7.00)	17.95 (1,57)	<.001	0.46
			Empathy	12.90 (6.56)	14.85 (8.59)	20.30 (2.50)	14.15 (8.12)	19.37 (1,57)	<.001	0.49
			Fantasy	13.20 (3.49)	13.57 (4.18)	12.50 (4.35)	14.64 (5.37)	1.11 (1,57)	.23	0.05
			Perspective	13.30 (5.74)	12.93 (6.06)	19.10 (3.21)	13.93 (7.43)	6.58 (1,57)	.02	0.24
	**Quality of life** ^f^ **(grade 1-10)**			(n=21)	(n=25)	(n=21)	(n=25)			
		Informal caregivers		7.24 (1.58)	6.23 (1.75)	7.05 (1.77)	6.48 (1.58)	0.00	.97	0.00
	**Burden** ^f^ **(score 1-5)**									
		Informal caregivers		2.67 (1.11)	3.08 (1.13)	2.43 (0.98)	2.80 (0.96)	0.63	.43	0.02
	**Sense of competence (SSCQ)** ^**f,g**^ **(score 0-7)**									
		Informal caregivers		4.43 (1.25)	4.54 (1.56)	4.67 (1.06)	4.04 (1.49)	5.50	.02	0.11

^a^For all scores except distress, burden, and sense of competence, the higher score is the more favorable.

^b^Effect size (η^2^) is considered small at 0.01, medium at 0.06, and large at 0.14.

^c^ADQ: Approaches to Dementia Questionnaire

^d^For professional caregivers, score is 11-55 because they had one additional question compared to informal caregivers.

^e^ADKS: Alzheimer’s Disease Knowledge Scale

^f^These were questionnaires specifically relevant to informal caregivers; they were not applied in volunteers.

^g^SSCQ: Short Sense of Competence Questionnaire

For the primary outcome measures, we found a statistically significant effect on attitudes toward dementia (ADQ total score) with a large effect size (η^2^) and on its subscale, person-centered care among laypeople (informal caregivers and volunteers), with a large effect size. For both cases, both the experimental group and the control group increased in score. Differences in baseline scores were accounted for by adding these as a covariate in the analyses. We did not find outliers at baseline that could explain this result. For the remaining primary outcomes (knowledge about and attitudes toward dementia), we did not find statistically significant differences in the experimental or control group among laypeople and professionals.

There were 2 separate questions relating to attitudes toward dementia. The first question related to which disease people were most afraid of developing (diabetes, stroke, heart disease, dementia, cancer, depression, or influenza). At pretest, 31% of laypeople were most afraid of developing dementia; for professionals, this was 13%. At post-test, 46% of laypeople and 8% of professionals were most afraid of developing dementia. This indicates that after completing the course, laypersons were more afraid of developing dementia, whereas this fear decreased in professionals. The second question on attitudes toward dementia was “if you had a family member who was exhibiting confusion and memory loss, would you want the person to see a doctor to determine if the cause of the symptoms was Alzheimer’s disease or not?”. This was answered positively by all who completed the research, both at pretest and at post-test.

For the secondary outcome measures, we found statistically significant differences between the experimental and control group in the expected direction on several subscales of empathy: the distress subscale, empathy subscale, and perspective subscale. Although the scores on these scales remained largely the same in the control group at pretest and post-test, we witnessed a significant improvement in the experimental group, indicating that they felt less distressed in tense situations, had more empathy and concern for the well-being of other people, and were better able to understand situations and the actions of other people. On the other hand, we found a medium-sized, significant negative effect on sense of competence of informal caregivers, which declined in the experimental group compared to the control group, implying that participants in the course felt less competent to fulfill their care task after following the course. Post hoc analysis revealed that this decline in sense of competence was related to a higher age of the caregivers (*r*=–.34, *P*=.02).

## Discussion

The STAR training, an online e-learning course developed to skill/reskill informal caregivers, volunteers, and professional caregivers of persons with dementia, was evaluated in the Netherlands and the United Kingdom on its usefulness, user friendliness, and effectiveness. This evaluation was undertaken in an RCT, comparing participants who followed the STAR training to a waiting list control group. The evaluation results indicated that, in general, the 8 modules of the STAR training were positively/very positively valued with regard to usefulness and user friendliness. The course was considered easy to undertake and the material was considered well thought out. Participants indicated that the course made them feel more secure about their quality as a caregiver. Although all modules were assessed positively, some modules, such as modules 1 and 3, scored lower than others did. The content of these modules will need to be reviewed for future versions of STAR.

The results of the RCT in the Netherlands and the United Kingdom demonstrated a significant positive impact of the STAR training course on maintaining feelings of empathy among informal caregivers and volunteers. Also, an effect was found on a person-centered care approach; both the person-centered care approach and the total score on positive approaches toward dementia increased among laypeople in both the experimental and the control group. The sense of competence declined slightly in the informal caregivers who followed the course, which appeared to be related to the higher age of the caregivers. The decline in sense of competence is in contrast to the finding that a large number of the participants who followed the STAR training indicated that they felt more secure about their qualities as caregivers.

For professional caregivers, empathy improved among those who followed the course. This is an indication that after following the course they became better able to view situations from another’s perspective (eg, a person with dementia) and that they showed more sympathy and concern, which may help them to provide better care for people with dementia. No effects were found on knowledge about Alzheimer’s disease. This was likely because the selected instrument, the ADKS, mostly has factual questions on symptoms and prevalence, although the STAR course predominantly focused on informing caregivers how to deal and cope with the consequences of dementia. Therefore, positive outcomes related to dealing with dementia (eg, empathy and attitudes), rather than an increase in knowledge, were in-line with our expectations. On other outcome measures, such as quality of life and burden, no effects were found.

These results are in-line with recent research, which has also found beneficial effects from Internet-based interventions [7], among others on attitudes and empathy [10]. One of the explanations for the effectiveness of STAR is the opportunity to choose “personalized learning paths,” which make it possible for each individual caregiver to tailor the content of the training to their own needs and skill level by recommending which modules are most relevant to them. According to earlier research by Lustria et al [8], tailored computer-based health interventions lead to improved health in caregivers. Previous research also indicates that increasing empathy in caregivers is highly relevant because it increases the well-being of the person with dementia [25].

Review studies on the effectiveness of psychosocial and technology-driven interventions to support family caregivers show that interventions using a psychoeducational or psychotherapeutic approach appear to be among the most powerful psychosocial interventions to improve quality of life of persons with dementia and their caregivers, and delay patient institutionalization [26-28]. Nevertheless, many studies suffered from serious methodological problems, such as unclear randomization methods, inadequate power calculation, selectively reported outcomes, and no use of an intention-to-treat analysis [29-31]. In addition, interventions were difficult to compare because type and intensity varied [32]. STAR adds to this research by offering an RCT with clear randomization methods and adequate numbers for sufficient statistical power. Additionally, all outcomes of STAR are clearly reported.

A strength of this study is that the STAR training portal was tested in an RCT design in 2 countries. A limitation of the study was the high number of dropouts in the RCT (34% in the Netherlands, 53% in the United Kingdom), especially in the experimental group; 43% (Netherlands) and 64% (United Kingdom) dropped out of the study. It seems likely that participants in the control group were more motivated to participate in the post-test because participating in the questionnaires would offer them free access to the course afterward. Furthermore, given that the STAR training portal was still in late development at the time of testing, some errors occurred when people followed the course. Another limitation was the fact that the online communities were not used often by participants, making them less informative and supportive than originally anticipated. The communities only contained a small number of participants because access was limited to only those in the experimental group of the research. However, they are expected to become more active and supportive in the future. This expectation is based on the fact that the STAR website has recently been updated to show links to the community websites more clearly. Additionally, when STAR gets more users, more users will potentially visit these communities, making them more lively and, therefore, more interesting to use for other visitors. We found little use for the Learning Path Advisor in the United Kingdom compared to use of it in the Netherlands. One explanation for this could be that the British group consisted of more volunteers with less experience in dementia care, who may have been more interested to follow the entire course, whereas the Dutch group consisted largely of informal caregivers and the majority (79%) had been caring for a person with dementia for 2 years or longer. It is likely that these experienced caregivers tended to use the Learning Path Advisor more frequently to find out which modules would provide them with new information, taking into account the knowledge they already had.

In conclusion, based on the promising results of our study, especially the positive effects of the STAR training portal on empathy for both laypeople (informal caregivers and volunteers) and professionals, it is recommended to repeat the RCT on a larger scale and in more countries. STAR is currently available in Dutch and English; the basic and intermediate modules are available in Italian and Romanian, and some are available in Swedish as well.

The positive effects of STAR on attitudes and empathy of caregivers may contribute to appropriate and high-quality dementia care in the community now and in the future. Therefore, an Internet-based intervention such as STAR can be a very useful alternative for face-to-face education and support for caregivers/informal caregivers and low-/unschooled professionals [2,3] and at lower costs [9], thus providing a means to cope with the challenge of taking care of the growing number of people with dementia in our society.

## Appendix

Multimedia Appendix 1 Participant flowchart for CONSORT.

Multimedia Appendix 2 CONSORT checklist PDF.

## Abbreviations

ADKS: Alzheimer’s Disease Knowledge Scale
ADQ: Approaches to Dementia Questionnaire
DSM-IV: Diagnostics and Statistics Manual, IV (4th) Edition
EU: European Union
IRI: Interpersonal Reactivity Index
RCT: randomized controlled trial
SSCQ: Short Sense of Competence Questionnaire
STAR: Skills Training And Reskilling
USE: Usefulness, Satisfaction, and Ease of use
