# Supplementary material for: Web-Based STAR E-Learning Course Increases Empathy and Understanding in Dementia Caregivers: Results from a Randomized Controlled Trial in the Netherlands and the United Kingdom
Source: J Med Internet Res. 2015 Oct 30;17(10):e241. doi: 10.2196/jmir.4025 (PMC4642792; doi:10.2196/jmir.4025)
Supplement: Multimedia Appendix 1 [file jmir_v17i10e241_app1.pdf]

Flowchart STAR participants.
